# Supplementary material for: The Nef Protein of the Macrophage Tropic HIV-1 Strain AD8 Counteracts Human BST-2/Tetherin
Source: Viruses. 2020 Apr 18;12(4):459. doi: 10.3390/v12040459 (PMC7232323; doi:10.3390/v12040459)
Supplement: Supplementary file 1 [file viruses-12-00459-s001.zip › viruses-729678-for conversion-suppl/Table S1.pdf]

Name	Vector	Origin	Description
AD8(+)	pBR322	K. Strebel (NIAID, Bethesda, USA)	See reference (1).
AD8(-)	pBR322	K. Strebel (NIAID, Bethesda, USA)	See reference (1).
AD8(U_{del2})	pBR322	K. Strebel (NIAID, Bethesda, USA)	See reference (2).
AD8(+)ΔNef	pBR322	this study	Nef-deficient AD8(+). Nef frame shift mutation introduced using the primers 5'-GGCCT ACTGT AAGGG AAAGA ATAGA CACGA GCTGA GC-3' and 5'-GCTCA GCTCG TGTCT ATTCT TTCCC TTACA GTAGG CC-3', and the QuikChange II XL Site-Directed Mutagenesis Kit (Agilent Technologies, Wokingham, UK) according to the manufacturer's instructions.
AD8(-)ΔNef	pBR322	this study	Nef-deficient AD8(-). Nef frame shift mutation introduced as described for AD8(+)ΔNef.
AD8(U_{del2})ΔNef	pBR322	this study	Nef-deficient AD8(U_{del2}). Nef frame shift mutation introduced as described for AD8(+)ΔNef.
VSVG	pMDG	G. Towers (UCL, London, UK)	-
AD8 Nef wild type	pcDNA3.1(+)	this study	The Nef open reading frame (ORF) was amplified from AD8(+) and inserted into pcDNA3.1 using BamH1 and EcoR1 restriction sites.
AD8 Vpu	pcDNA3.1(+)	this study	The Vpu open reading frame (ORF) was amplified from AD8(+) and inserted into pcDNA3.1 using BamH1 and EcoR1 restriction sites.
AD8 Env	pcDNA3.1(+)	this study	The Env open reading frame (ORF) was amplified from AD8(-) and inserted into pcDNA3.1 using BamH1 and EcoR1 restriction sites.
HIV-1 Rev	pBC12/CMV	G. Towers (UCL, London, UK)	-
AD8 Nef E160A_LL164/165AA	pcDNA3.1(+)	this study	The mutations LL164/165AA and E160A were consecutively introduced into AD8 Nef wild type using the primers 5'-CAATA AAGGA GAGAA CAACT GCGCG GCACA CCCTA TGAGC CAGCA TGGA-3' and 5'-TCCAT GCTGG CTCAT AGGGT GTGCC GCGCA GTTGT TCTCT CCTTT ATTG-3', and 5'-AGGCC AATAA AGGAG CGAAC AACTG CGCGG C-3' and 5'-GCCGC GCAGT TGTTC GCTCC TTTAT TGGCC T-3', respectively, and the QuikChange II Site-Directed Mutagenesis Kit (Agilent Technologies) according to the manufacturer's instructions.
AD8 Nef DD174/175RA	pcDNA3.1(+)	this study	The mutation was introduced into AD8 Nef wild type using the primers 5'-CAACA CTTCT CTCTC CGTGG CACGC ATTCC ATGCT GGCTC ATA-3' and 5'-TATGA GCCAG CATGG AATGC GTGCC ACGGA GAGAG AAGTG TTG-3', and the QuikChange II Site-Directed Mutagenesis Kit (Agilent Technologies) according to the manufacturer's instructions.

AD8 Nef E160A_LL164/165AA_ DD174/175RA	pcDNA3.1(+)	this study	The mutation DD174/175RA was introduced into AD8 Nef E160A_LL164/165A using the primers listed above and the QuikChange II Site-Directed Mutagenesis Kit (Agilent Technologies) according to the manufacturer's instructions.
AD8 Nef G2A	pcDNA3.1(+)	this study	The mutation was introduced into AD8 Nef wild type using the primers 5'-CTCGG ATCCA AGATG GCTGG CAAGT GGTCA AAA-3' and 5'-TTTTG ACCAC TTGCC AGCCA TCTTG GATCC GAG-3', and the QuikChange II Site-Directed Mutagenesis Kit (Agilent Technologies) according to the manufacturer's instructions.
NL4.3 Nef	pcDNA3.1(+)	this study	The Nef open reading frame (ORF) was amplified from pNL4.3 (provided by C. Jolly, UCL, London, UK) and inserted into pcDNA3.1 using BamH1 and EcoR1 restriction sites.
AD8-NL4.3 Nef	pcDNA3.1(+)	this study	The N-terminal AD8 Nef domain was excised from AD8 Nef wild type using BamH1 and Bgl2 and ligated into NL4.3 Nef digested with the same restriction enzymes.
NL4.3-AD8 Nef	pcDNA3.1(+)	this study	The N-terminal NL4.3 Nef domain was excised from NL4.3 Nef using BamH1 and Bgl2, and ligated into AD8 Nef wild type digested with the same restriction enzymes.
AD8 Nef IRES GFP	pCG	this study	The ORF was inserted into pCG using Xba1 and Mlu1 restriction sites.
NL4.3 Nef IRES GFP	pCG	modified in this study (originally obtained from F. Kirchhoff, University of Ulm, Germany)	The ORF was inserted into pCG using Xba1 and Mlu1 restriction sites.
JR-CSF Nef IRES GFP	pCG	F. Kirchhoff (University of Ulm, Germany)	-
SF2 Nef IRES GFP	pCG	F. Kirchhoff (University of Ulm, Germany)	-
CH077 Nef IRES GFP	pCG	F. Kirchhoff (University of Ulm, Germany)	-
CH198 Nef IRES GFP	pCG	F. Kirchhoff (University of Ulm, Germany)	-
2693BA Nef IRES GFP	pCG	F. Kirchhoff (University of Ulm, Germany)	-
OMRCA Nef IRES GFP	pCG	modified in this study (originally obtained from F. Kirchhoff, University of Ulm, Germany)	The ORF was inserted into pCG using Xba1 and Mlu1 restriction sites.
13127K2 Nef IRES GFP	pCG	F. Kirchhoff (University of Ulm, Germany)	-
RBF168 Nef IRES GFP	pCG	F. Kirchhoff (University of Ulm, Germany)	-
7312 Nef IRES GFP	pCG	modified in this study (originally obtained from	The ORF was inserted into pCG using Xba1 and Mlu1 restriction sites.

		F. Kirchhoff, University of Ulm, Germany)	
NL4.3 Nef_stop IRES GFP	pCG	F. Kirchhoff (University of Ulm, Germany)	-

1. **Theodore TS**, **Englund G**, **Buckler-White A**, **Buckler CE**, **Martin MA**, **Peden KW**. 1996. Construction and characterization of a stable full-length macrophage-tropic HIV type 1 molecular clone that directs the production of high titers of progeny virions. AIDS Res Hum Retroviruses **12**:191–194.

2. **Schubert U**, **Clouse KA**, **Strebel K.** 1995. Augmentation of virus secretion by the human immunodeficiency virus type 1 Vpu protein is cell type independent and occurs in cultured human primary macrophages and lymphocytes. J Virol **69**:7699–7711.
